# Tip-Viscid Electrohydrodynamic Jet 3D Printing of Composite Osteochondral Scaffold

**DOI:** 10.3390/nano11102694

**Published:** 2021-10-13

**Authors:** Kai Li, Dazhi Wang, Fangyuan Zhang, Xiaoying Wang, Hairong Chen, Aibing Yu, Yuguo Cui, Chuanhe Dong

**Affiliations:** 1 School of Mechanical Engineering and Mechanics, Ningbo University, Ningbo 315211, China; zhangfangyuan@nbu.edu.cn (F.Z.); chenhairong@nbu.edu.cn (H.C.); yuaibing@nbu.edu.cn (A.Y.); cuiyuguo@nbu.edu.cn (Y.C.); 2 State Key Laboratory of Digital Manufacturing Equipment and Technology, Huazhong University of Science and Technology, Wuhan 430074, China; 3 Key Laboratory for Micro/Nano Technology and System of Liaoning Province, Dalian University of Technology, Dalian 116024, China; d.wang@dlut.edu.cn; 4 School of Civil and Environmental Engineering, Ningbo University, Ningbo 315211, China; wangxiaoying1@nbu.edu.cn; 5 Research Center of Intelligent Industrial Robot, Chinese Academy of Sciences, Jinan 250100, China; dongchuanhe1024@163.com

**Keywords:** 3D printing, electrohydrodynamic jet, thermal field, fluid viscosity, osteochondral scaffold

## Abstract

A novel method called tip-viscid electrohydrodynamic jet printing (TVEJ), which produces a viscous needle tip jet, was presented to fabricate a 3D composite osteochondral scaffold with controllability of fiber size and space to promote cartilage regeneration. The tip-viscid process, by harnessing the combined effects of thermal, flow, and electric fields, was first systematically investigated by simulation analysis. The influences of process parameters on printing modes and resolutions were investigated to quantitatively guide the fabrication of various structures. 3D architectures with high aspect ratio and good interlaminar bonding were printed, thanks to the stable fine jet and its predictable viscosity. 3D composite osteochondral scaffolds with controllability of architectural features were fabricated, facilitating ingrowth of cells, and eventually inducing homogeneous cell proliferation. The scaffold’s properties, which included chemical composition, wettability, and durability, were also investigated. Feasibility of the 3D scaffold for cartilage tissue regeneration was also proven by in vitro cellular activities.

## 1. Introduction

Cartilage is an essential tissue in humans and animals, playing an important role in the structure and function of the ear, nose, and interosseous joint [1]. However, it is difficult for the cartilage to repair itself when damaged, due to the lack of nerve, lymphatic, and blood supply [2,3,4]. Cartilage tissue can suffer from many kinds of injuries and the repairing process involves a complex therapeutic pathology [5]. Repairing the cartilage to restore its normal tissue and function is challenging for medical workers. It has been reported that tissue engineering researches based on in-vitro cell culture has found significance in exploring the inducement of damaged cartilage and implanting chondrocytes [6,7,8]. In the two-dimensional (2D) cell culture technique, cells grow on a flat substrate, such as dishes or plates [9,10,11]. However, these methods are no longer sufficient in many cases. Most cells reside in complex three-dimensional (3D) microenvironments, where complex factors, such as cell-extracellular matrix (ECM) interaction, cell–cell interaction, biochemical factors, and shear stress are induced by fluid flow [12,13]. It has been demonstrated that cells behave more natively when cultured in 3D environments [14]. Thus, tissue engineering scaffold is becoming an ideal medium for 3D cell culture [15,16,17]. 3D tissue engineering of scaffold has been verified as an effective approach for tissue regeneration [18].

A variety of technological approaches have been used to construct bioengineered 3D culturing scaffolds [19]. Conventional fabrication technologies, such as solvent casting [20], phase separation [21] and freeze-drying [22], lack capabilities to precisely control scaffold characteristics, such as porosity and pore size. A printing technology that could properly control a scaffold’s structural features has been proposed [23,24,25]. Unlike conventional manufacturing methods, printing makes it possible to rapidly fabricate complex 3D structures on demand, those without dyes and with simple processes [26,27,28]. Extrusion printing can pattern biomaterials in the form of printable inks. The biomaterial fiber is extruded through a needle and deposited onto a print surface. This “pushing” printing technique is simple and of low cost [29,30]. Several 3D printing methods utilize this approach, including direct ink writing (DIW) [31] and fused deposition modelling (FDM) [32]. The attainable printing resolution mainly relies on the needle inner size. Thus, the major drawback of these extrusion printing technologies is its large fiber size, when compared to the scale of living cartilage cells [33,34]. The printed scaffolds are limited in regard to cellular attachment and tissue formation because of insufficient micro/nanostructured contact inducement.

Electrohydrodynamic jet printing (E-Jet) with the application of electrohydrodynamically induced flow forms a jet, which is based on a “pulling” force rather than a “pushing” force, showing a distinct advantage of high resolution [35,36]. In contrast to DIW and FDM, whose printed fiber diameter is close to needle inner size, E-Jet printing can obtain a much smaller fiber. It can also be controlled to achieve the desired fiber diameter, ranging from nanoscale to microscale, by properly choosing process parameters like flow rate, applied voltage, and printing height, among others [37]. This provides an innovative way to fabricate scaffolds, in which its fibers are close to the scale of living cells.

In recent explorations, melt-based E-jet printing method was proposed to fabricate a 3D scaffold. A heating module, such as a medium circulating heating module or a resistive heating module, is required to melt the solid biopolymers into printable melts [38]. The feature sizes of melt-based E-jet printed fibers are normally between sub-microns and tens of microns, close to cell size, and offer a desired approach to mimic the 3D microstructure of native ECM. The melt-based E-jet printing method was employed to manufacture a lattice or tubular 3D biopolymer scaffold with microscale fibers [39]. Previous studies have demonstrated that the viscosity of melted biopolymer ink has a significant effect on the scaffold morphology [34]. This phenomenon illustrates that it is difficult to fabricate a composite biopolymer scaffold with various biopolymers or functional particles due to the existing differences in glass conversion temperature. Another important fabricating form is the solution-based E-Jet printing process. Instead of melting a biopolymer as the high viscous printable ink, solution-based E-Jet printing employs a low viscous solution to fabricate a biopolymer scaffold, enabling easy incorporation of various biomaterials into the printed scaffold [40]. However, one of the major challenges for solution-based E-Jet printing is to achieve rapid evaporation of the solvent to prevent the printed fiber from collapsing. It is also hard to construct high aspect ratio structures as it is possible for the low viscosity solution to flow on the substrate.

To address these challenges, a modified TVEJ printing method was presented to print a low viscosity PCL/PVP ink in a highly tip-viscid jetting form to fabricate a composite osteochondral scaffold by harnessing the combined effects of thermal, flow, and electric fields. Theoretical and experimental results showed that a single microneedle could produce high viscosity ink with local biopolymer concentration much higher than its initial state on exposure to thermal effects, and various printing modes and structure resolutions can be controlled flexibly by adjusting the rate of solvent evaporation. To rationally select parameters for this novel printing strategy, the influences of printing parameters were investigated to quantitatively guide ink viscosity, jetting stability, and printed resolution. By selecting optimal processing parameters, the capability of adjusting jetting size in reproducible and predictable viscosity further enabled us to build architectures with varying aspect ratio and desired interlaminar bonding. Control of the process could provide controllability of fiber size and space during fabrication of the composite osteochondral scaffold, which facilitated ingrowth of cells, eventually inducing homogeneous cell proliferation in whole composite scaffold. In order to overcome the hydrophobic intrinsic property of PCL, hydrophilic PVP was added to fabricate the osteochondral scaffold. The various physical properties of the scaffold, including composite component, wettability, and durability in water were studied. To show feasibility as a scaffold for cartilage tissue regeneration, the fabricated scaffolds were studied in in vitro cellular activities, including cell proliferation and activities using Murine MC3T3-E1 Subclone14 cartilage cells.

## 2. Materials and Methods

### 2.1. Printing Set-Up

**Figure 1:**
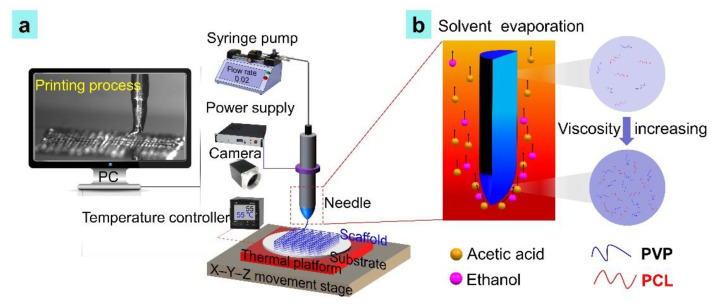
(**a**) Schematic diagram showing the TVEJ printing process; (**b**) local biopolymer concentration and tip viscosity increasing during printing.

Figure 1a shows a schematic illustration of the TVEJ experimental set-up. A quartz needle (New Objective, Inc., Littleton, MA, USA) was fixed on an aluminum conductive fixture and connected to a glass syringe (Hamilton, Reno, NV, USA) through a metallic hose to supply the prepared PCL/PVP composite ink. The syringe was fixed to a syringe pump (Harvard Apparatus, Holliston, MA, USA). A high voltage power supply (Tianjin Dong Wen High Voltage Power Supply Co., Ltd., Tianjin, China) was connected to the aluminum conductive fixture. A thermal platform was placed on the movement stage. The resistance heaters in the thermal platform were employed to provide steady temperature. A temperature-controller was used to adjust the temperature. A camera (Point Grey, Richmond, Canada) was employed to observe jetting status.

### 2.2. PCL/PVP Composite Ink Composition and Preparation

PCL particles, PVP powder, and acetic acid, and Triton X-100 were purchased from Yuanye Bio-Technology Co., Ltd. (Shanghai, China), Aladdin Industrial Corporation (Shanghai, China), and Adamas Reagent Co., Ltd. (Shanghai, China). PEG, ethanol, and Triton X-100 were purchased from Kemiou Chemical Reagent Co., Ltd. (China). 9.1 wt.% of PCL particle (Mw = 800,000) was dissolved in acetic acid. The PCL ink was obtained after the PCL particles completely dissolved. For preparation of PVP ink, 11.7 wt.% of PVP powder (Mw = 1,300,000) was dissolved in ethanol. Then 0.4 wt.% of PEG and 1.7 wt.% of Triton X-100 were orderly added to the solution. The PVP ink was then obtained. The two inks were mixed at room temperature to obtain printable PCL/PVP composite ink.

### 2.3. Evaluation of Composite Ink

Surface tension, an important parameter for TVEJ printing, was measured using an automatic surface tensiometer. The maximum measurement value was 1–500 mN m^−1^ and resolution was 0.01 mN m^−1^ (K100C, Kruess Scientific Instrument, Hamburg, Germany). The mean value of surface tension was calculated after being tested 10 times.

### 2.4. 3D Printing Procedure

The prepared PCL/PVP composite ink was loaded on to a syringe. A syringe pump pushed the ink into a quartz needle at a constant flow rate using hydrodynamic force. This process was done for 3 min to remove air bubbles in the needle. Subsequently, the thermal platform started to work, retaining a certain temperature around the needle. Local biopolymer concentration occurred at the needle tip and the ink viscosity increased due to solvent evaporation (acetic acid, ethanol) under a thermal effect (Figure 1b). An electric shearing force provided by the high voltage power supply acted on the high viscosity ink. A fine jet was formed at the needle tip by harnessing the combined effects of thermal, flow, and electric fields. Printing paths of the PCL/PVP composite osteochondral scaffold were designed by LabVIEW procedure. Jetting behaviors were observed in real-time using a camera.

### 2.5. Theoretical Analysis of the Tip-Viscid E-Jet Printing Process

During the TVEJ printing, the thermal convection in the PCL/PVP composite at the needle tip can be expressed by the following equation [41]:

(1) $$\rho C_{p}u\cdot \nabla T+\nabla q=Q+Q_{p}+Q_{vd}$$

where *ρ* is the density (kg m^−3^), *C_p_* is the specific heat capacity (J (kg·K)^−1^), ***u*** is the velocity vector (m s^−1^), *T* is the absolute temperature (K), ***q*** is the heat flux by conduction (W m^−2^), *Q* contains heat sources other than viscous dissipation (W m^−3^), *Q_p_* is the work done by pressure changes.

The fluids above the thermal platform, including the PCL/PVP composite and air, are a continuous medium. Thus, Fourier’s law of thermal convection, which is proportional to the temperature gradient, can be expressed by an equation:

(2) q=-k∇T

where *k* is the thermal conductivity (W (m·K)^−1^).

(3) $$Q_{p}=a_{p}T(\frac{\partial p}{\partial t}+u\cdot \nabla p)$$

(4) $$a_{p}=-\frac{1}{\rho }\frac{\partial p}{\partial T}$$

(5) $$Q_{vd}=\tau :\nabla \mu$$

where *α_p_* is the coefficient of thermal expansion (1 K^−1^), *p* is the pressure (Pa), ***τ*** is the viscous stress tensor (Pa).

The PCL/PVP composite and air are based on the laws of conservation of momentum, mass, and energy. The conservation of mass in those fluids can be expressed by a continuity equation:

(6) $$\frac{\partial p}{\partial t}+\nabla (\rho u)=0$$

The conservation of momentum can be expressed by a vector equation:

(7) $$\rho \frac{\partial u}{\partial t}+\rho (u\cdot \nabla )u=\nabla (-pI+\tau )+F$$

The conservation of energy, formulated in terms of temperature, can be expressed by an equation:

(8) $$\rho C_{p}(\frac{\partial T}{\partial t}+(u\cdot \nabla )T)=-(\nabla \cdot q)+\tau :S-{\frac{T}{\rho }\frac{\partial \rho }{\partial T}|}_{P}(\frac{\partial p}{\partial t}+(u\cdot \nabla )p)+Q$$

(9) $$S=\frac{1}{2}(\nabla u+{\left(\nabla u\right)}^{T})$$

(10) $$\tau =2\mu S-\frac{2}{3}u(\nabla u)I$$

where ***u*** is the velocity vector (m s^−1^), *p* is pressure (Pa), ***τ*** is the viscous stress tensor (Pa), ***F*** is the volume force vector (N m^−3^), *q* is the heat flux vector (W m^−2^), ***S*** is the strain-rate tensor.

### 2.6. Cell Culture

The 12th generation of Murine MC3T3-E1 Subclone14 cells were cultured in a DMEM (Hyclone, Logan, UT, USA) medium with 10% fetal bovine serum (FBS, MINHAI, Shenzhen, China), 100 units mL^−1^ penicillin and 100 μg mL^−1^ streptomycin (Hyclone, Logan, UT, USA) at 37 °C. Prior to cell seeding, the osteochondral scaffolds were sterilized in 75% alcohol (4 h). And osteochondral scaffolds were air dried. 20 µL cell suspensions with density of 5 × 10^5^ cell mL^−1^ were drop-seeded on two sides of osteochondral scaffold. The fresh medium was added after incubated at 37 °C for 1  h and 5 *v*/*v*% CO_2_ atmosphere. And the fresh medium was changed termly according to cell culture plan.

### 2.7. Cell Viability

Dead/live staining was performed to evaluate cell viability and attachment after cell seeding 1, 2, 3 days, respectively. The characterizations of the cell-scaffold were treated with Calcein-AM, propidium iodide (PI), and Hochest 33,258 staining solution for 30 min. Cell viability on the PCL/PVP composite scaffolds were observed randomly by a fluorescence microscope. Cell viabilities were estimated after culturing for 1, 2, 3 days, respectively. The cells on scaffolds were observed by field emission scanning electron microscopy. For SEM, the cell-scaffold construct was fixed using glutaraldehyde and dehydrated using an aqueous solution containing 50, 70, 90, and 100% ethanol concentration. Data were presented as mean ± standard deviations.

## 3. Results and Discussion

### 3.1. Evaluation of the Printable PCL/PVP Composite Ink

The novel TVEJ printing method presented in this study was developed to fabricate composite osteochondral scaffolds under the combined effects of thermal, flow, and electric fields. Low viscous ink undergoes solvent evaporation at the needle tip under effects of a thermal field. As the solvent evaporates during printing, the diameter of the fiber decreases and its viscosity gradually increases with time due to a local higher biopolymer concentration (see Figure 1b). This viscosity gradient enables the creation of high-resolution 2D patterning and a high aspect ratio 3D structure with desired interlaminar bonding by changing the temperature of the thermal field. After most of the solvent evaporates, the rigidity of the jetted fiber changes from fluid-like to solid-like, promoting the structure feature retention of the deposited 3D structures, especially for 3D hierarchical composite osteochondral scaffolds.

**Figure 2:**
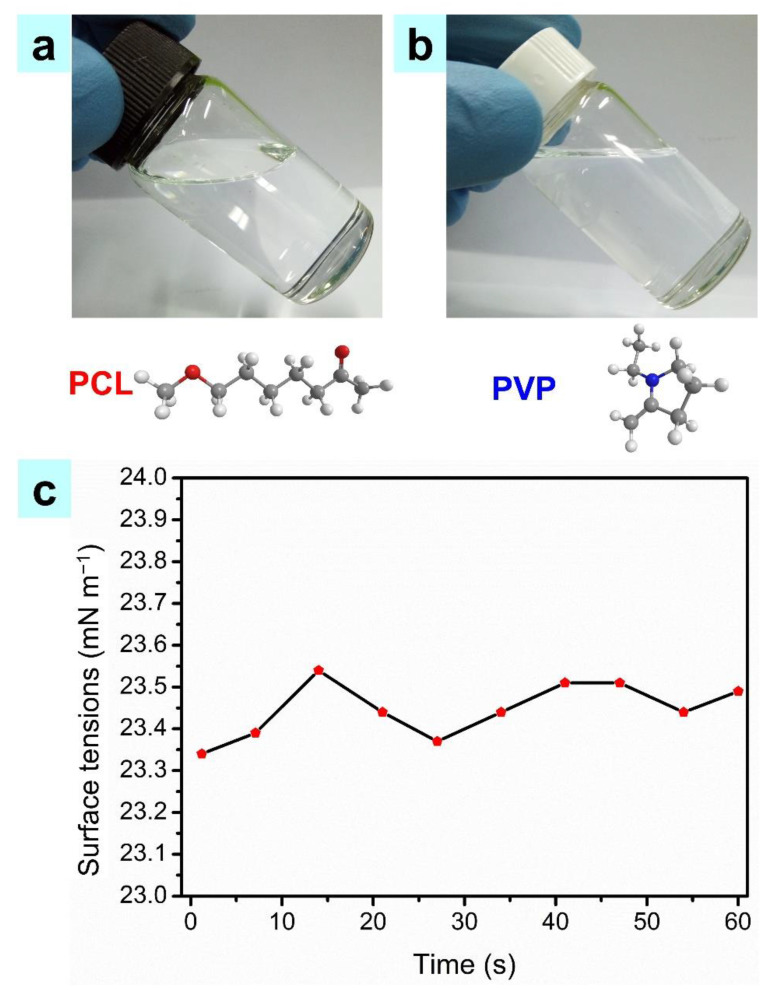
(**a**) The PCL ink; (**b**) The PVP ink; (**c**) The measured surface tensions of the composite ink.

Figure 2a,b show the images of the prepared PCL ink and the PVP ink. The two inks maintained stable dispersion conditions and were highly transparent. They were mixed in a certain proportion to produce the composite PCL/PVP ink. The surface tension of the composite ink was measured multiple times at 20 °C. As shown in Figure 2c, the mean surface tension was 23.5 mN m^−1^. This indicated that the applied voltage threshold of the printing PCL/PVP ink was small because a low surface tension solution only required a small electric field force (*F_e_*) to induce a jet [42,43].

### 3.2. The Process of Tip-Viscid Jetting

**Figure 3:**
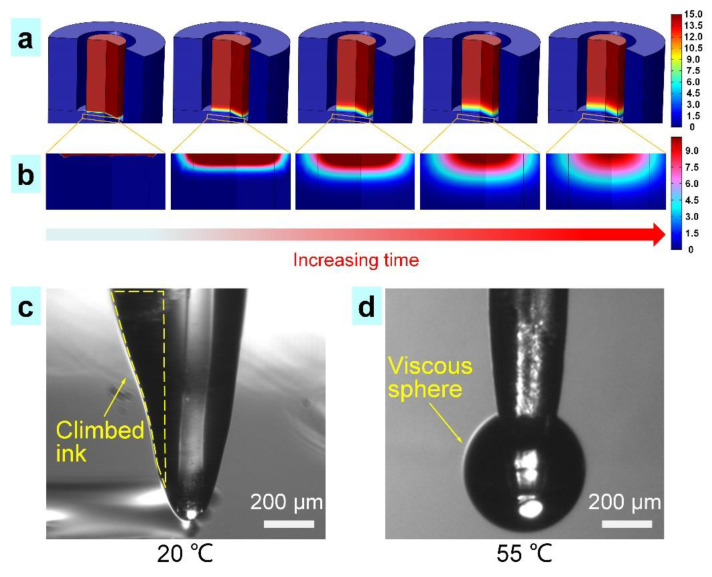
The process of tip-viscid. (**a**) The simulation results of the solvent content at 55 °C (unit, mol L^−1^); (**b**) The solvent content in the jet; (**c**) The ink climbed along the needle without a thermal effect (at 20 °C); (**d**) The ink transformed into a high viscous sphere with a thermal field (at 55 °C), suspending at the needle tip.

A theoretical model was developed in COMSOL software to analyze the solvent evaporation and ink viscosity generated in the TVEJ printing process. According to the proportion of the PCL/PVP composite ink, the solvent content was calculated to be 15 mol L^−1^, and the temperature was set at 55 °C. Figure 3a,b show the change of solvent content in the TVEJ printing model. As the time increased, the solvent content at the needle tip decreased accordingly. Most of the ethanol and acetic acid is evaporated at 55 °C and the ink viscosity further increased at the needle tip, resulting in a high viscous state, which was beneficial in maintaining a stable continuous fine jet.

The solvent content at the cross-section profile of the needle tip was obtained from Figure 3a,b. The final solvent content was found to be 3 mol L^−1^, which was four times lower than the initial content of 15 mol L^−1^. Ink behavior at the needle tip with and without thermal effects was observed. As shown in Figure 3c, the low viscosity ink climbed along the needle because of capillarity (see yellow frame) without thermal effects. When subject to thermal effects (temperature of 55 °C), the composite ink transformed into a highly viscous sphere, suspended at the needle tip (Figure 3d). This novel tip-viscid process in E-Jet printing not only produces a stable and fine jet (sub-micrometer), but also avoids clogging because it only occurs at the needle tip.

**Figure 4:**
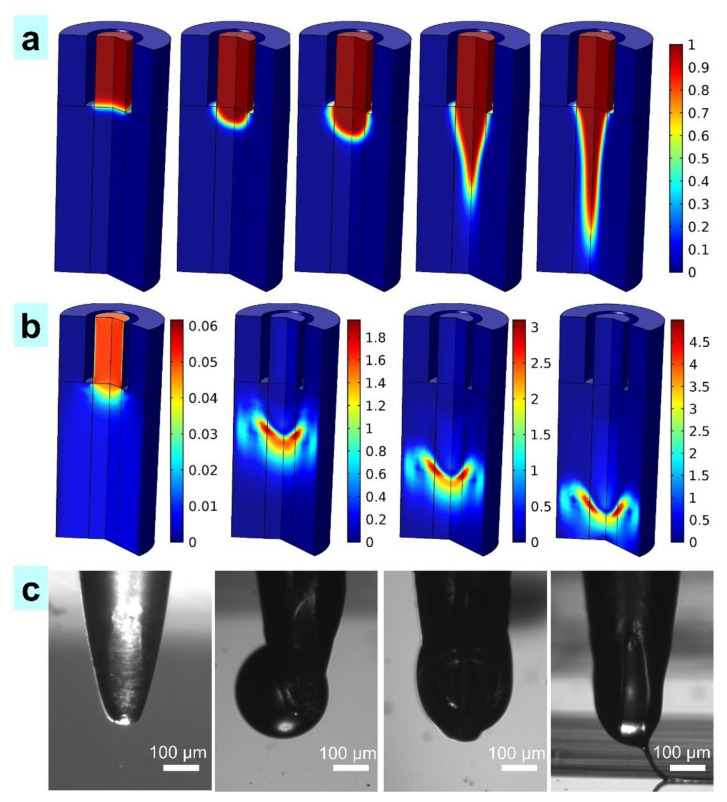
The formation of a viscous jet. (**a**) The simulation results of the viscous jet (0 µs, 2 µs, 4 µs, 6 µs and 8 µs, respectively); (**b**) Jet speed, 2 µs, 4 µs, 6 µs, and 8 µs, respectively (unit, mm s^−1^); (**c**) The experimental process of the viscous jet (scale, 100 µm).

Figure 4a shows the morphologies of the simulated TVEJ printing while the temperature, applied voltages, and flow rate were 55 °C, 800 V, and 6 × 10^−11^ m^3^ s^−1^, respectively. The red region shows the PCL/PVP composite ink defined as the liquid phase, and the blue region the air phase. The mobile ions in the high viscosity PCL/PVP composite ink were supplied by a high potential and accumulated persistently, and this process lead to an intense *F_e_* on the pendant viscous sphere. The TVEJ printing technology utilized *F_e_* to induce high viscosity PCL/PVP composite ink, forming a stable and fine jet at the needle tip, while the electrostatic stresses overcame the surface tension. Figure 4b shows the jet speed (*S_j_*) in the printing process, and it could be concluded that *S_j_* reached the maximum with the formation of the jet. In this study, the initial *S_j_* was set at 0.05 mm s^−1^, and the final *S_j_* was able to reach 5 mm s^−1^ and hit the substrate quickly. This was because the *F_e_* lead to the accumulation of mobile ions in the high viscosity composite ink to regions near the surface of the pendant viscous meniscus. As the PCL/PVP composite ink was continuously supplied, the pressure and speed of ink inside the needle were maintained. The effect of tangential *F_e_* was enhanced, and the fiber diameter decreased with continuous voltage. Figure 4c shows the experimental evolution process of the viscous jet, which was consistent with the simulation.

### 3.3. Diverse Modes and Resolutions in TVEJ Printing

**Figure 5:**
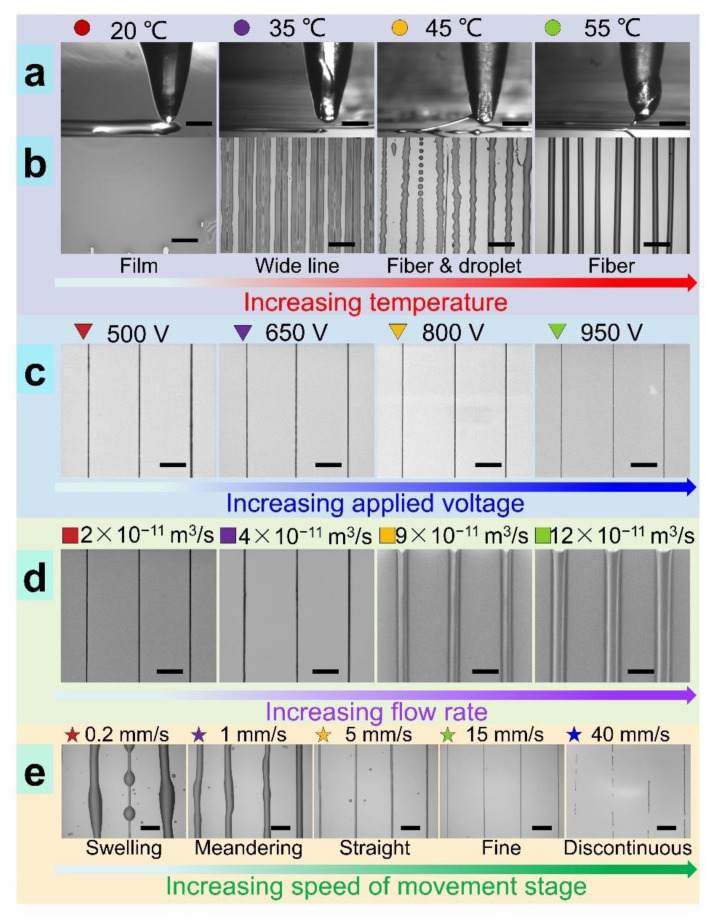
Diverse modes and resolution in TVEJ printing by varying selections of printing parameters. (**a**) Jet behaviors under different temperatures; (**b**) Diverse modes under different temperatures; (**c**,**d**) The printed resolution under different applied voltages and flow rates; (**e**) Diverse modes under different *S_m_*. (all scale, 200 µm).

As shown in Figure 5a, the new TVEJ printing method can produce droplets, continuous and discontinuous fibers far finer than the needle’s inner size; furthermore, a thin film, 2D complex patterns (e.g., square, spiral, pentacle), and 3D architectures could be fabricated. In order to control various printing modes, parameter matching for each mode needs to be quantitatively identified. As illustrated in Figure 5b, the low viscosity droplets eject from the needle tip and overlap each other when printing at 20 °C, resulting in thin films. Increasing temperature, wide lines, and fiber appeared in turn. The transition process during different printing modes could be continuous, enabling fabrication of various non-linear structures and fiber diameters in a controllable manner. Figure 5c shows the effect of applied voltage on fiber size. It can be seen that finer fiber can be obtained when a higher voltage is applied. The tangential *F_e_* on the cone surface improved as the applied voltage increased; *S_j_* toward the substrate was faster, resulting in a shorter and thinner jet.

Figure 5d shows that the effect of flow rate on fiber size was obvious. The increase in flow rate caused the increase in flow rate output, resulting in large size fiber. It was also found that the speed of movement stage (*S_m_*) had a significant influence on the printing mode and fiber size. When *S_m_* was much lower than the *S_j_*, TVEJ was unable to print linear and aligned microstructures, even if the movement stage was moving linearly (see first two in Figure 5e). The swelling and meandering characteristic resulted from the speed difference between *S_j_* and *S_m_*. *S_j_* was 5 mm s^−1^, while the applied voltage and flow rate were constant (see simulation results in Figure 4b). When *S_j_* (5 mm s^−1^) exceeded *S_m_* (0.2, 1 mm s^−1^), a large amount of viscous composite ink would be printed onto the substrate, resulting in printing characteristic of swelling and meandering. The fiber diameter and their spacing could be tuned by *S_m_*. When *S_j_* was close to *S_m_* (5 mm s^−1^), the printed fiber became straight due to the effect of the mechanical drawing force induced by moving. Linear arrays of microscale fibers with quantitative spacing were fabricated to demonstrate the relationship between *S_m_* and the printed feature. When *S_j_* was far less than *S_m_* (15, 40 mm s^−1^), an unstable jet and discontinuous printed lines appeared, even if the spacing was uniform. This was because the volume of the remaining viscous composite ink in the needle was insufficient to maintain stable jet formation when a larger *S_m_* was selected. It was also observed that a cavity usually appeared inside the needle, which also could interrupt the continuous jetting. It can be concluded that the optimized *S_m_* was close to *S_j_* in the TVEJ printing process.

### 3.4. Printing of 3D Structures and Composite Scaffolds

**Figure 6:**
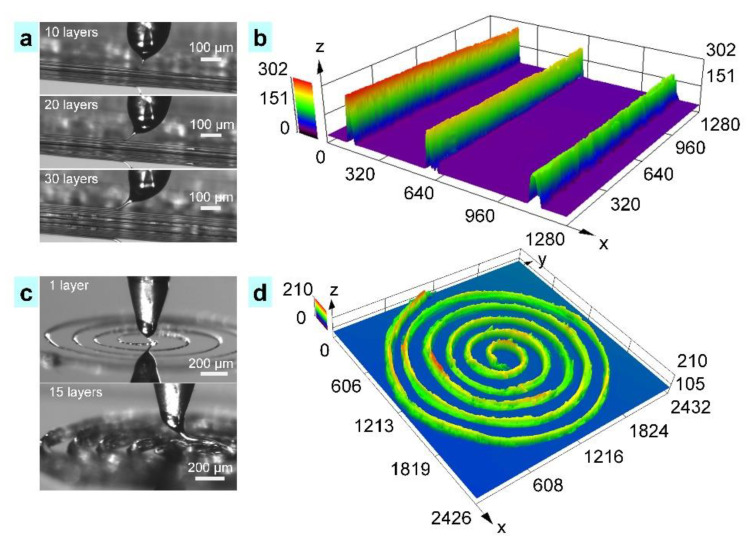
TVEJ printing of 3D structures with varying aspect ratio and desired interlaminar bonding. (**a**) The printing process of perpendicular walls with 10, 20, and 30 layers and its interlaminar bonding; (**b**) 3D profile of the perpendicular walls (unit, µm); (**c**) Fabricating process of a 3D spiral structure in continuous printing sequences; (**d**) 3D profile of a spiral structure (unit, µm).

The capability of adjusting fiber size in reproducible and predictable viscosity is important to print 3D architectures with varying aspect ratio and desired interlaminar bonding. As shown in Figure 6, 3D structures could be printed by overlaying printing fibers repetitively; and the height of the structure could be controlled by the number of overlaid printing sequences without any significant change in lateral dimensions. Compared to traditional melt E-Jet printing, which was prone to crack and interlaminar defects, the 3D structures prepared by TVEJ printing technology has a smooth interface, thanks to the flexible adjustable ink viscosity. Figure 6a presents the fabricating process of 3D perpendicular walls under 10, 20, and 30 layers, respectively. As demonstrated in Figure 6b, fibers with uniform size and appropriate viscosity were stacked layer by layer; and fibers with certain viscosity bonded to each other, showing good interlaminar combination. Figure 6b shows the 3D profile of the perpendicular walls. When 30 layers were printed, interlamination of the perpendicular wall was found to be completely overlapped and the aspect ratio was about 23. Figure 6c shows the fabricating process of the 3D spiral structure in continuous printing sequences. The diameter of inside circle was about 400 µm. After printing 20 layers, the height of the 3D spiral structure was 210 µm (Figure 6d). During the printing in Figure 6, the temperature, applied voltage, flow rate, and *S_m_* were 55 °C, 800 V, 7 × 10^−11^ m^3^ s^−1^, and 2 mm s^−1^, respectively. This successful fabrication of various 3D structures proved that the new TVEJ technology has the ability to construct microstructures with high aspect ratio and great interlaminar bonding.

**Figure 7:**
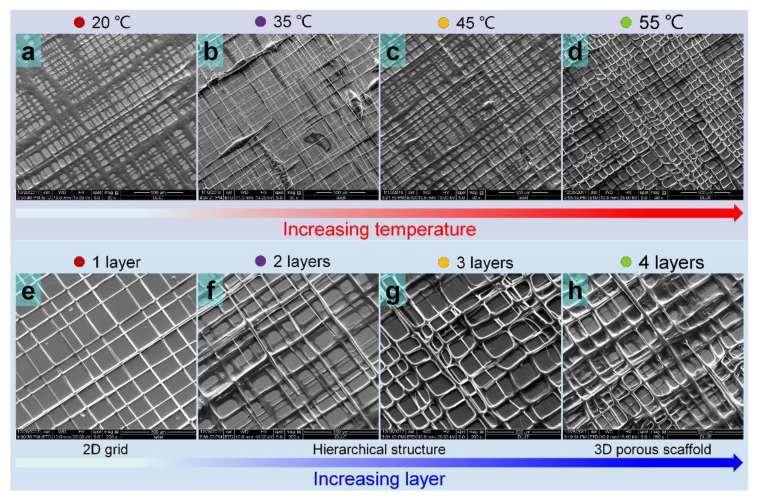
SEM images of TVEJ printed composite osteochondral scaffolds. (**a**–**d**) Printing a four-layer scaffold at different temperatures; (**e**–**h**) Printed scaffolds with a different layer at 55 °C.

In Figure 7a–d, the composite osteochondral scaffolds (four layers) printed at various temperatures are shown. Other printing parameters, including applied voltage, flow rate, and *S_m_* were set at 900 V, 4 × 10^−11^ m^3^ s^−1^, and 8 mm s^−1^, respectively. When printing at room temperature (20 °C), a unique structure consisting of wide lines and macropores was fabricated. This might be considered as preparation of a thick film containing many macropores. Although four layers were printed, with a low printing temperature and a low viscosity cone at the needle tip, a unique hierarchical structure was not attained (35 °C, 45 °C, Figure 7b,c). This result was consistent with the conclusion in Figure 5b. At a higher printing temperature (55 °C, Figure 7d), continuous and fine fibers were obtained, and hierarchical osteochondral scaffolds was obtained easily due to the significantly stable jetting behavior and faster solidification of the landing fiber. The influence of printing layers on the structural characteristics of fabricated composite osteochondral scaffolds is described in Figure 7e–h. A 2D grid structure was fabricated on printing only one layer (Video S1). The 3D hierarchical composite osteochondral scaffolds were easier to fabricate, while porous characteristics were obtained with increase in printed layers (Video S2).

### 3.5. Property of the Printed Scaffold

**Figure 8:**
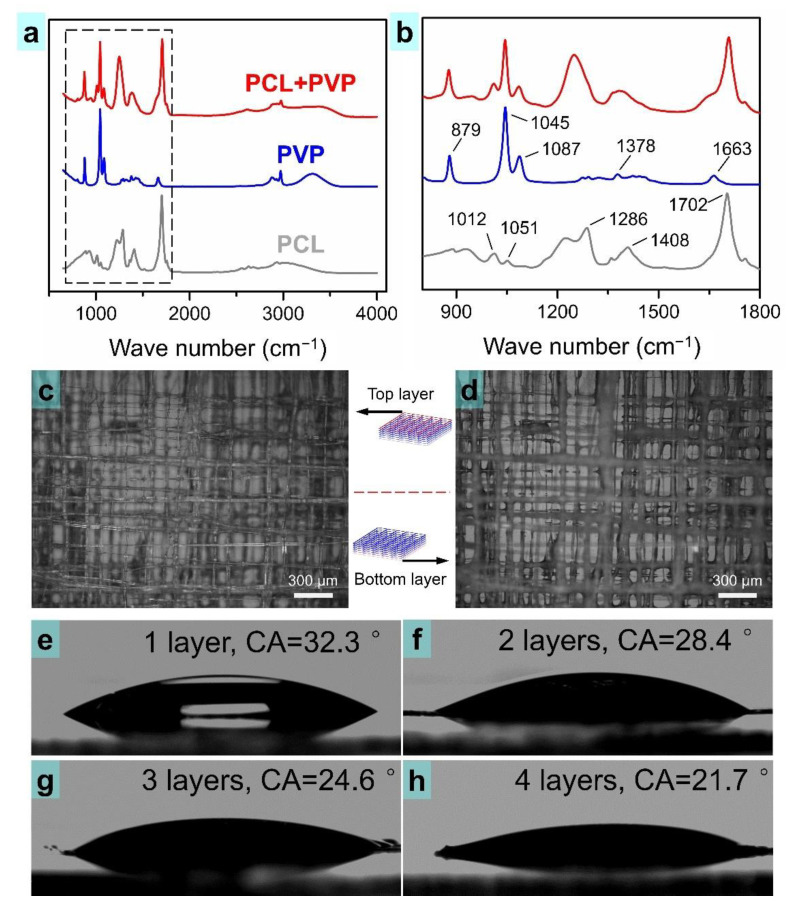
(**a**,**b**) FTIR spectra and its characteristic peaks. (**c**,**d**) Soaking scaffold in deionized water for 3 days; (**e**–**h**) Contact angle measurements of the composite scaffold with 1, 2, 3, and 4 layers, respectively.

The novel TVEJ printing technology involved multi-field coupling processes, and the printable PCL/PVP composite ink was subjected to high temperature and high applied voltage at the nanoliter volume level. In order to verify that the PCL and PVP were well-preserved in the composite osteochondral scaffold, Fourier transform infrared (FTIR) spectra were performed (Figure 8a). Figure 8b (gray line) shows the PCL scaffold spectrum. The stretching vibration at 1702 cm^−1^ demonstrated a peak of C=O; the stretching vibration at 1408 cm^−1^ and 1286 cm^−1^ exhibited CH_2_ bending modes and COO– vibrations [44]. Figure 8b (blue line) shows the PVP scaffold spectrum. The C=O stretching vibrations were at 1663 cm^−1^. CH_2_ bending modes was observed at 1378 cm^−1^ and an intense sharp peak of C–N vibrations were exhibited at 1045 cm^−1^ [45]. A dominant peak of the PCL scaffold and PVP scaffold obviously existed in the PCL/PVP composite scaffold (red line). The FTIR spectra results illustrated that a chemical bonding or physical interaction almost never happened during printing of the PCL/PVP composite scaffold, though strong multi-field coupling processes existed in the TVEJ printing technology.

Wettability is one of the crucial factors influencing cell attachment to scaffolds [46]. PCL has been proved to be a biocompatible polymer suitable for many tissue engineering applications. However, its hydrophobic intrinsic property is a hindering factor to some extent. Accordingly, fabrication of a composite osteochondral scaffold comprising of PCL and PVP could be a reliable strategy to overcome this constraint. There was one key point—PVP is hydrophilic and durability of the PCL/PVP composite scaffold in cell culture medium is crucial. In order to verify this problem, the PCL/PVP composite scaffold was soaked in deionized water for three days and its structural features observed. As shown in Figure 8c,d, the overall shape of the scaffold and its fibers could be kept stable, in both top and bottom layers. The surface roughness of the fibers in the composite scaffold increased, thank to PVP dissolving partially. Contact angle (CA) was measured to evaluate the surface wettability of the composite scaffold with different layers (Figure 8e–h). The CA of the PCL/PVP composite osteochondral scaffolds, from 1 to 4 layers, were 32.3°, 28.4°, 24.6°, and 21.7°, respectively. On printing more layers, the composite osteochondral scaffold became more hydrophilic.

### 3.6. Cell Morphology on the Scaffolds

**Figure 9:**
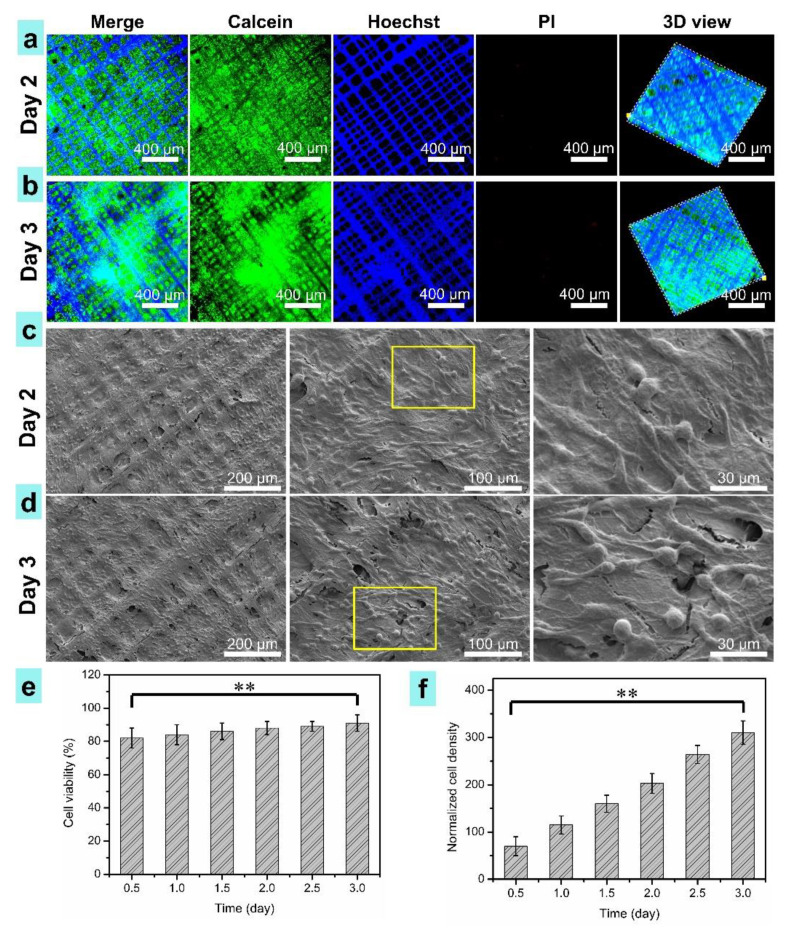
Characterization of cartilage cells on the printed PCL/PVP composite osteochondral scaffolds. (**a**,**b**) The LSCM fluorescent images, scale bars, 400 μm; (**c**,**d**) SEM observations of cell morphology; (**e**) Cell viability; (**f**) Normalized cells densities. ** indicate statistical significance of *p* < 0.01 (*n* = 4).

Figure 9 shows the evaluation results of cartilage cells’ activity and distribution on the printed osteochondral scaffolds after different days of cell culture. Dead/live staining of the 3D microtissue of cells grown on the printed scaffold was conducted for 1, 2, and 3 days of culture. Live cells, dead cells, and cell nuclei were stained with Calcein-AM, PI, and Hoechst 33258. The LSCM fluorescent images are shown in Figure 9a,b, exhibiting that the cells well attached and proliferated on the printed osteochondral scaffolds. The cell density in the composite scaffold for three-day culture (Figure 9b, Video S4) was apparently higher than that of two days (Figure 9a, Video S3). The 3D view in Figure 9a,b and Videos S3 and S4 proved that the cells were totally attached to the osteochondral scaffolds and the cells’ ingrowth height was about 25 μm.

As shown in Figure 9c,d, cartilage cells attached well on the surface of the printed PCL/PVP composite osteochondral scaffolds. The surface of the scaffolds was covered with cells after three-day culture (Figure 9c). As shown in Figure 9e, the cell viabilities were about 84%, 88%, and 91% after cell culture of 1, 2, and 3 days, respectively. An increase in cell viability was found in the composite scaffold with more culture time. The normalized cartilage cells’ densities were measured termly (Figure 9f). It was indicated that cell growth was significantly enhance with increase in days of cell culture. After three days of culture, the cells were about four times as dense as when just seeded. These results indicate that the printed composite osteochondral scaffold has great biocompatibility and feasibility for cartilage tissue regeneration.

## 4. Conclusions

A PCL/PVP composite osteochondral scaffold was fabricated by a novel TVEJ printing method that produced a viscous tip jet after local biopolymer concentration under the combined effects of thermal, flow, and electric fields. This modified printing method showed that ink viscosity was much higher than its initial state on exposure to thermal effects. In addition, various printing modes and resolutions were obtained by adjusting the rate of solvent evaporation. The influence of printing parameters was investigated to quantitatively guide the fabrication of various structures. 3D architectures with high aspect ratio and well interlaminar bonding were printed by selecting optimal processing parameters. The composite osteochondral scaffold with controllability of fiber size and space was fabricated, facilitating ingrowth of cells, and eventually inducing homogeneous cell proliferation in the entire scaffold. Hydrophilic PVP ink was added to PCL ink to fabricate the osteochondral scaffold to reduce the hydrophobic intrinsic property of PCL. Various physical properties of the scaffold (component, wettability, and durability) were studied. Furthermore, nanoscale pores exposed on the printed fibers could point towards meaningful enhancement of in vitro cellular responses. To show feasibility of the scaffold for cartilage tissue regeneration, the fabricated scaffold was investigated in in vitro cellular activities, including cell proliferation. Based on the results, we can state that this novel TVEJ printing technology can successfully fabricate composite osteochondral scaffolds, which could serve as a tissue regeneration medium biomaterial of immense potential for cartilage tissue repair.

## Supplementary Materials

The following are available online at https://www.mdpi.com/article/10.3390/nano11102694/s1, Video S1: Printing of composite osteochondral scaffold with one layer, Video S2: Printing of multi-layers composite scaffold, Video S3: The cartilage cell on printed scaffold after two days of cell culture, Video S4: The cartilage cell on printed scaffold after three days of cell culture.

## References

[1] Lam J., Clark E.C., Fong E.L.S., Lee E.J., Lu S., Tabata Y., Mikos A.G. (2016). Evaluation of cell-laden polyelectrolyte hydrogels incorporating poly(L-Lysine) for applications in cartilage tissue engineering. Biomaterials.

[2] Lu C., Yeh T., Yeh C., Fang Y.D., Sung L., Lin S., Yen T., Chang Y., Hu Y. (2014). Regenerating Cartilages by Engineered ASCs: Prolonged TGF-beta 3/BMP-6 Expression Improved Articular Cartilage Formation and Restored Zonal Structure. Mol. Ther..

[3] Hu X., Man Y., Li W., Li L., Xu J., Parungao R., Wang Y., Zheng S., Nie Y., Liu T. (2019). 3D Bio-Printing of CS/Gel/HA/Gr Hybrid Osteochondral Scaffolds. Polymers.

[4] Zhu D., Wang H., Trinh P., Heilshorn S.C., Yang F. (2017). Elastin-like protein-hyaluronic acid (ELP-HA) hydrogels with decoupled mechanical and biochemical cues for cartilage regeneration. Biomaterials.

[5] Sophia Fox A.J., Bedi A., Rodeo S.A. (2009). The basic science of articular cartilage: Structure, composition, and function. Sports Health.

[6] Moroni L., Burdick J.A., Highley C., Lee S.J., Morimoto Y., Takeuchi S., Yoo J.J. (2018). Biofabrication strategies for 3D in vitro models and regenerative medicine. Nat. Rev. Mater..

[7] Sorushanova A., Delgado L.M., Wu Z., Shologu N., Kshirsagar A., Raghunath R., Mullen A.M., Bayon Y., Pandit A., Raghunath M. (2019). The Collagen Suprafamily: From Biosynthesis to Advanced Biomaterial Development. Adv. Mater..

[8] Dong R., Ma P.X., Guo B. (2020). Conductive biomaterials for muscle tissue engineering. Biomaterials.

[9] Giulitti S., Magrofuoco E., Prevedello L., Elvassore N. (2013). Optimal periodic perfusion strategy for robust long-term microfluidic cell culture. Lab Chip.

[10] Halldorsson S., Lucumi E., Gomez-Sjoberg R., Fleming R.M.T. (2015). Advantages and challenges of microfluidic cell culture in polydimethylsiloxane devices. Biosens. Bioelectron..

[11] Li J., Wei J., Liu Y., Liu B., Liu T., Jiang Y., Ding L., Liu C. (2017). A microfluidic design to provide a stable and uniform in vitro microenvironment for cell culture inspired by the redundancy characteristic of leaf areoles. Lab Chip.

[12] Webber M.J., Appel E.A., Meijer E.W., Langer R. (2016). Supramolecular biomaterials. Nat. Mater..

[13] Lutolf M.P., Hubbell J.A. (2005). Synthetic biomaterials as instructive extracellular microenvironments for morphogenesis in tissue engineering. Nat. Biotechnol..

[14] Murphy S.V., Atala A. (2014). 3D bioprinting of tissues and organs. Nat. Biotechnol..

[15] Li K., Wang D., Zhao K., Song K., Liang J. (2020). Electrohydrodynamic jet 3D printing of PCL/PVP composite scaffold for cell culture. Talanta.

[16] Lai Y., Li Y., Cao H., Long J., Wang X., Li L., Li C., Jia Q., Teng B., Tang T. (2019). Osteogenic magnesium incorporated into PLGA/TCP porous scaffold by 3D printing for repairing challenging bone defect. Biomaterials.

[17] Yan Y., Chen H., Zhang H., Guo C., Yang K., Chen K., Cheng R., Qian N., Sandler N., Zhang Y.S. (2019). Vascularized 3D printed scaffolds for promoting bone regeneration. Biomaterials.

[18] Diomede F., D’Aurora M., Gugliandolo A., Merciaro I., Orsini T., Gatta V., Piattelli A., Trubiani O., Mazzon E. (2018). Biofunctionalized scaffold in bone tissue repair. Int. J. Mol. Sci..

[19] Chen F.M., Liu X. (2016). Advancing biomaterials of human origin for tissue engineering. Prog. Polym. Sci..

[20] Deliormanli A.M., Atmaca H. (2020). Effect of pore architecture on the mesenchymal stem cell responses to graphene/polycaprolactone scaffolds prepared by solvent casting and robocasting. J. Porous Mater..

[21] Ko Y.G. (2020). Formation of oriented fishbone-like pores in biodegradable polymer scaffolds using directional phase-separation processing. Sci. Rep..

[22] Norouzi S.K., Shamloo A. (2019). Bilayered heparinized vascular graft fabricated by combining electrospinning and freeze drying methods. Mater. Sci. Eng. C-Mater. Biol. Appl..

[23] Youssef A., Hollister S.J., Dalton P.D. (2017). Additive manufacturing of polymer melts for implantable medical devices and scaffolds. Biofabrication.

[24] Mondschein R.J., Kanitkar A., Williams C.B., Verbridge S.S., Long T.E. (2017). Polymer structure-property requirements for stereolithographic 3D printing of soft tissue engineering scaffolds. Biomaterials.

[25] Pizzicannella J., Diomede F., Gugliandolo A., Chiricosta L., Bramanti P., Merciaro I., Orsini T., Mazzon E. (2019). 3D Printing PLA/Gingival Stem Cells/EVs Upregulate miR-2861 and -210 during Osteoangiogenesis Commitment. Int. J. Mol. Sci..

[26] Truby R.L., Lewis J.A. (2016). Printing soft matter in three dimensions. Nature.

[27] Zhang Y., Zhang F., Yan Z., Ma Q., Li X., Huang Y., Rogers J.A. (2017). Printing, folding and assembly methods for forming 3D mesostructures in advanced materials. Nat. Rev. Mater..

[28] MacDonald E., Wicker R. (2016). Multiprocess 3D printing for increasing component functionality. Science.

[29] Qin Z., Compton B.G., Lewis J.A., Buehler M.J. (2015). Structural optimization of 3D-printed synthetic spider webs for high strength. Nat. Commun..

[30] Lind J.U., Busbee T.A., Valentine A.D., Pasqualini F.S., Yuan H., Yadid M., Park S.J., Kotikian A., Nesmith A.P., Campbell P.H. (2017). Instrumented cardiac microphysiological devices via multimaterial three-dimensional printing. Nat. Mater..

[31] Therriault D., White S.R., Lewis J.A. (2003). Chaotic mixing in three-dimensional microvascular networks fabricated by direct-write assembly. Nat. Mater..

[32] Zein I., Hutmacher D.W., Tan K.C., Teoh S.H. (2002). Fused deposition modeling of novel scaffold architectures for tissue engineering applications. Biomaterials.

[33] Li K., Wang D., Wang Q., Song K., Liang J., Sun Y., Madoua M. (2018). Thermally assisted electrohydrodynamic jet high-resolution printing of high-molecular weight biopolymer 3D structures. Macromol. Mater. Eng..

[34] He J., Xia P., Li D. (2016). Development of melt electrohydrodynamic 3D printing for complex microscale poly (epsilon-caprolactone) scaffolds. Biofabrication.

[35] Kim B.H., Onses M.S., Lim J.B., Nam S., Oh N., Kim H., Yu K.J., Lee J.W., Kim J.H., Kang S.K. (2015). High-resolution patterns of quantum dots formed by electrohydrodynamic jet printing for light-emitting diodes. Nano Lett..

[36] Park J.U., Lee S., Unarunotai S., Sun Y., Dunham S., Song T., Ferreira P.M., Alleyene A.G., Paik U., Rogers J.A. (2010). Nanoscale, electrified liquid jets for high-resolution printing of charge. Nano Lett..

[37] Onses M.S., Sutanto E., Ferreira P.M., Alleyne A.G., Rogers J.A. (2015). Mechanisms, capabilities, and applications of high-resolution electrohydrodynamic jet printing. Small.

[38] Wei C., Dong J. (2014). Hybrid hierarchical fabrication of three-dimensional scaffolds. J. Manuf. Process..

[39] Hochleitner G., Jungst T., Brown T.D., Hahn K., Moseke C., Jakob F., Dalton P.D., Groll J. (2015). Additive manufacturing of scaffolds with sub-micron filaments via melt electrospinning writing. Biofabrication.

[40] Lei Q., He J., Li D. (2019). Electrohydrodynamic 3D printing of layer-specifically oriented, multiscale conductive scaffolds for cardiac tissue engineering. Nanoscale.

[41] Bird R.B., Stewart W.E., Lightfoot E.N. (2007). Transport Phenomena.

[42] Bae J., Lee J., Hyun Kim S. (2017). Effects of polymer properties on jetting performance of electrohydrodynamic printing. J. Appl. Polym. Sci..

[43] Rosell-Llompart J., Grifoll J., Loscertales I.G. (2018). Electrosprays in the cone-jet mode: From Taylor cone formation to spray development. J. Aerosol Sci..

[44] Charernsriwilaiwat N., Rojanarata T., Ngawhirunpat T., Opanasopit P. (2016). Aligned electrospun polyvinyl pyrrolidone/poly ε-caprolactone blend nanofiber mats for tissue engineering. Int. J. Nanosci..

[45] Wang B., Wu S., Ahmad Z., Li J., Chang M.-W. (2018). Co-printing of vertical axis aligned micron-scaled filaments via simultaneous dual needle electrohydrodynamic printing. Eur. Polym. J..

[46] Ranella A., Barberoglou M., Bakogianni S., Fotakis C., Stratakis E. (2010). Tuning cell adhesion by controlling the roughness and wettability of 3D micro/nano silicon structures. Acta Biomater..

